# Continuous tobacco smoking increases mortality in diffuse large B-cell lymphoma but not follicular lymphoma, a Finnish population-based study

**DOI:** 10.2340/1651-226X.2025.44776

**Published:** 2025-12-17

**Authors:** Taina Reunamo, Erika Alanne, Toni Mikkola, Antti Karlsson, Antti Ellonen, Tarja Laitinen, Maarit Bärlund, Pia Österlund, Heikki Minn, Sirpa Leppä, Sirkku Jyrkkiö, Eetu Heervä

**Affiliations:** a Department of Oncology, Turku University Hospital and University of Turku, Turku, Finland; b Wellbeing services county of Southwest Finland, Turku, Finland; c Western Finland Cancer Centre, Turku, Finland; d Department of Oncology and Tays Cancer Center, Tampere University Hospital, Wellbeing Services County of Pirkanmaa, Tampere, Finland; e University of Turku, Turku, Finland; f Pihlajalinna Oyj, Tampere, Finland; g Institute for Molecular Medicine Finland, University of Helsinki, Helsinki, Finland; h Faculty of Medicine and Health Technology, University of Tampere, Tampere, Finland; i Department of Oncology and Research Programs Unit, Faculty of Medicine, University of Helsinki, Helsinki, Finland; j Comprehensive Cancer Centre, Helsinki University Hospital, Helsinki, Finland

**Keywords:** Tobacco smoking, diffuse large B-cell lymphoma, follicular lymphoma, real word data

## Abstract

**Background and purpose:**

Tobacco smoking was prognostic in B-cell lymphomas in the pre-rituximab era, but the association with modern treatment, stage, subtypes, and survival outcomes remains unclear.

**Patient/material and methods:**

All patients with diffuse large B-cell lymphoma (DLBCL) and follicular lymphoma (FL) from Turku and Tampere University Hospitals 2009–2019 were identified. Population-based data from electronic medical records included demographics, tumour histology, Ann Arbor staging, and treatments. Smoking status was extracted with a deep learning-based natural language processing algorithm. Kaplan–Meier overall survival (OS) estimates and adjusted hazard ratios (HRs) were calculated.

**Results:**

With a median follow-up of 96 months, 1,258 patients with DLBCL and 529 with FL were included. In DLBCL, the 5-year OS rate was 61%, 53%, and 45% among never, former, and persistent smokers, respectively. Persistent smoking remained an independent prognostic factor for shorter OS, HR 1.27 (95% confidence interval 1.10–1.60) after adjustment for comorbidities and completed treatment. The prognosis of FL was indolent with no difference in OS regardless of smoking status, with 5-year OS rates of 79%, 75%, and 74% among never, former, and persistent smokers, respectively. Smokers were younger at diagnosis, while other baseline demographics were similar. No differences in the systemic therapy use were observed between the different smoking categories in both FL and DLBCL.

**Interpretation:**

Overall and lymphoma-specific mortality is increased in persistent smokers with DLBCL compared with never smokers. Smoking prevention and cessation support remains of utmost importance.

## Introduction

Diffuse large B-cell lymphoma (DLBCL) is an aggressive lymphoid B-cell cancer and the most common lymphoma entity [1]. The standard DLBCL treatment is immunochemotherapy (ICT) containing the anti-CD20 antibody rituximab with a combination of cyclophosphamide, doxorubicin, vincristine, and prednisolone (R-CHOP regimen). In selected cases, dose-intensifications and consolidation radiotherapy may be offered [2–5]. R-CHOP regimen is considered curative, offering a complete response in 73%–82% of patients and resulting in 5-year overall survival (OS) rates of 60%–70% [6–10]. Reduced dose R-mini-CHOP is well- tolerated and has yielded clinically meaningful disease control in the elderly and frail patients [11]. Substantial improvements in survival rates compared with the R-CHOP regimen have not been clearly shown, regardless of intensive research with novel agents [12–14].

The more indolent-course follicular lymphoma (FL) is the second most common lymphoma subtype, with 5-year OS rates being up to 93% [1, 15]. The treatment strategies for FL vary according to clinical presentation from observation of asymptomatic patients to CD20 immunotherapy alone, or ICT for patients needing more aggressive treatment [16].

Data on post-diagnosis survival in DLBCL and FL relative to tobacco smoking are scarce. Retrospective questionnaire-based studies assessing this relationship were mostly conducted before the rituximab era [17–21]. Studies included heterogeneous groups of non-Hodgkin lymphomas and showed an impaired OS in patients with the most pack years smoked compared to never smokers. Subgroup analysis of patients with FL suggested that smoking impairs their OS [18, 20, 22]. while among patients with DLBCL, no differences were reported [17–20]. Only one study about smoking and survival during the rituximab era has been published: Among Australian patients with FL, smoking increased overall and lymphoma-specific mortality compared to never smokers in a dose-dependent manner [22]. In addition to these reports, smoking has also been suggested to increase the risk of developing FL [22–26], especially in women [25,26], but whether smoking increases the incidence of DLBCL is debatable [24–26].

Even though the literature is suggestive for a link between smoking status and outcome in B-cell lymphoma, a research gap exists. Population-based OS in B-cell lymphomas from Finland has been reported as low as 50%, clearly suggesting that treatment intensity and precise B-cell lymphoma diagnosis must be considered [9–10, 27]. Multiple smoking-related comorbidities may confound the lymphoma-specific outcomes [21, 28], along with other lifestyle factors [17–19, 29].

The aim of the current study was to analyse the impact of smoking status on crude and demographic/treatment-adjusted overall and lymphoma-specific survival (LSS) in patients with DLBCL and FL population-based.

## Patients/material and methods

### Study permissions

The study was approved by the Finnish Social and Health Data Permit Authority ‘Findata’ (THL529_14.02.00_2022). According to Finnish legislation, patients’ informed consent is not required for the secondary use of health and social data for register-based research. Local Institutional Review Board permissions and informed consent were not required for retrospective use of health records. Administrative permission was obtained from Turku University Hospital (T132/2019) and Tampere University Hospital (R19582, 1575/2021).

Findata maintains a secure data analysis and storaBaseline demographics in DLBCL accordingge environment (https://findata.fi/en/). The datasets were combined, and the personal data of study patients were processed and analysed in this secure and highly regulated environment. After anonymisation, only the aggregated summary statistics were released.

### Study population

The study population consisted of all patients with DLBCL and FL, diagnosed histologically during 2009–2019, identified at two Finnish University Hospitals, Turku and Tampere (catchment populations 480,000 and 540,000 inhabitants, respectively). These university hospitals cover all active treatments in their regions, while neighbouring smaller regions may refer additional selected lymphoma patients for radiotherapy, high-dose chemotherapy, or autologous stem cell transplantation.

Both hospitals have electronic medical record systems covering pathology, ICD-10 diagnoses, surgery, radiotherapy, and systemic anticancer treatments. Additionally, both hospitals have ‘data lakes’ in which data from different health care software systems can be combined and reorganised using patients’ unique social security number.

These two hospital data lakes were combined in a Findata analysis platform. Then, dates and causes of death were obtained from nationwide registry Statistics Finland at the cut-off of December 2022. Patients diagnosed at autopsy (*n* = 24) were excluded. Nine patients had both DLBCL and FL simultaneously and were classified as DLBCL.

### Data collection

To determine the smoking status, a natural language processing model as described previously was used [30, 31]. In short, all sentences specific to tobacco smoking were extracted from the medical narrative using a rule-based algorithm. The sentences were then further classified into three classes: persistent, former or never smoker. The deep learning network classifies all sentences at the patient level covering the entire observational period (2009–2019) and ultimately classifies the patient either as a never, former, or persistent smoker based on probability logic. Former smokers include patients who quit before or after cancer diagnosis. We reported earlier accuracy of 0.92, 0.78, and 0.92 for never, former, and persistent smokers, respectively [27].

Clinical variables collected included all ICD-10 codes, ECOG (Eastern Cooperative Oncology Group) performance status, and body mass index (BMI). Comorbidities were assessed using ICD-10 codes for the Charlson Comorbidity Index [32], where an index of 0 means lack of comorbidities. Ann Arbor stage (I–IV) was obtained directly from medical records.

Electronic chemotherapy charts were available from 2009 in Turku, but from 2012 in Tampere, with some missing information. The exact regimen could not be specified among 86 (7%) patients with DLBCL and 47 (9%) with FL. Total radiation doses (Gy) and target organ of radiotherapy were available. A radiotherapy dose of at least 30 Gy for DLBCL [3, 4] or 24 Gy for FL [16] was considered as a part of curative intent treatment and lower doses as palliative care.

### Statistics

Baseline demographics between never, former, and persistent smokers were compared with the Bonferroni-adjusted Chi-square method. OS was defined from the histological lymphoma diagnosis to death and LSS to death from lymphoma or leukaemia (C81-85 and C91-97), while other deaths were censored. Median survival and 5-year survival rates were estimated with the Kaplan–Meier method and hazard ratios (HRs) with the 95% confidence interval (CI) were calculated with Cox regression. The Cox proportional hazards assumption was assessed visually for crossing of the survival curves and no violations were observed. Median follow-up was calculated with the reverse Kaplan–Meier method. Missing data was apparently not random (Supplementary Table 2), and was forced into the multivariable model as ‘not available’. All statistical analyses were performed with SPSS version 27 (IBM Armonk, NY).

## Results

### DLBCL population

A total of 1,258 patients with DLBCL were identified. Datasets from Tampere and Turku were comparable (Supplementary Table 1), but Tampere had more missing data regarding smoking status (30% vs. 13%), stage (27% vs. 8%), and systemic regimens given (12% vs. 0%). Patients from Tampere had better ECOG performance status (ECOG 0–1 in 67% vs. 59%) but more comorbidities (57% vs. 47%) compared to patients from Turku.

Once combined (Supplementary Table 2), the median age of the cohort was 69 years, 76% of patients were aged over 60 years, 54% were male, and Ann Arbor stage was III–IV in 67% of the patients. Systemic therapy was initiated to 909 patients, ICT for at least six cycles for 489, and best supportive care or radiotherapy only in 349, whereas treatment was unclassified among 86 patients. Smoking status was available for 978 (78%) patients with DLBCL. Compared to DLBCL patients with known smoking status (Supplementary Table 2), those with unknown smoking status were typically older women (26% vs. 42% >75 years) receiving best supportive care or with unknown treatment information.

### Baseline demographics and treatment of DLBCL according to smoking status

Among patients with DLBCL, 563 (58%) were never, 229 (23%) former, and 186 (19%) persistent smokers. At diagnosis (Table 1), males smoked more frequently. Persistent smokers were younger at diagnosis compared to never smokers (age over 60 years 66% vs. 74%, and over 75 years 13% vs. 30%). Ann Arbor stage and ECOG performance status were equally distributed between the groups. Persistent smokers had more comorbidities than never smokers.

**Table 1:** Baseline demographics in DLBCL according to smoking status.

	Never smoker *n* = 563 (58%)	Former smoker *n* = 229 (23%)	Persistent smoker *n* = 186 (19%)	*p*-value
Age, years				
Median (IQR, range)	68.4 (60**–**77, 14**–**95)	68.3 (62**–**75, 29–97)	64.3 (58**–**71, 15**–**92)	
≤ 60 years	146 (26)	47 (21)	64 (34)	0.006
> 60 years	417 (74)	182 (79)	122 (66)	
≤ 75 years	392 (70)	175 (76)	162 (87)	< 0.001
> 75 years	171 (30)	54 (24)	24 (13)	
Sex				
Female	296 (53)	68 (30)	63 (34)	< 0.001
Male	267 (47)	161 (70)	123 (66)	
Ann Arbor stage				
I	89 (18)	32 (16)	28 (17)	0.82
II	82 (17)	29 (14)	20 (12)	
III	85 (17)	34 (17)	31 (19)	
IV	242 (49)	108 (53)	84 (52)	
Not available	65	26	23	
ECOG PS				
0–1	369 (73)	158 (76)	122 (74)	0.77
2–4	134 (27)	50 (24)	43 (26)	
Not available	60	21	21	
Charlson Comorbidity Index				
0	269 (48)	98 (43)	80 (43)	0.02
1	146 (26)	46 (20)	41 (22)	
2–6	148 (26)	85 (37)	65 (35)	
BMI				
Median (IQR)	26.4 (23.4**–**29.4)	27.6 (24.6**–**30.6)	25.6 (21.8**–**29.4)	0.27
BMI ≤ 22 kg/m^2^	67 (14)	13 (6)	27 (17)	0.005
BMI > 22 kg/m^2^	420 (86)	190 (94)	131 (83)	
Not available	76	26	28	

BMI: Body mass index; ECOG PS: Eastern Cooperative Oncology Group performance status; IQR: interquartile range.

Statistical testing only with known data.

No differences for systemic treatment or radiotherapy proportions were seen based on smoking status (Supplementary Table 3). Among never, former, and persistent smokers, 447 (79%), 183 (80%), and 135 (73%) were fit for systemic treatment, respectively, and 58%, 57%, and 59% completed six cycles of ICT. No differences were observed in the systemic regimens, number of cycles, or consolidation/palliative radiotherapy use between the different smoking groups.

### Smoking status and survival in DLBCL

**Figure 1:**
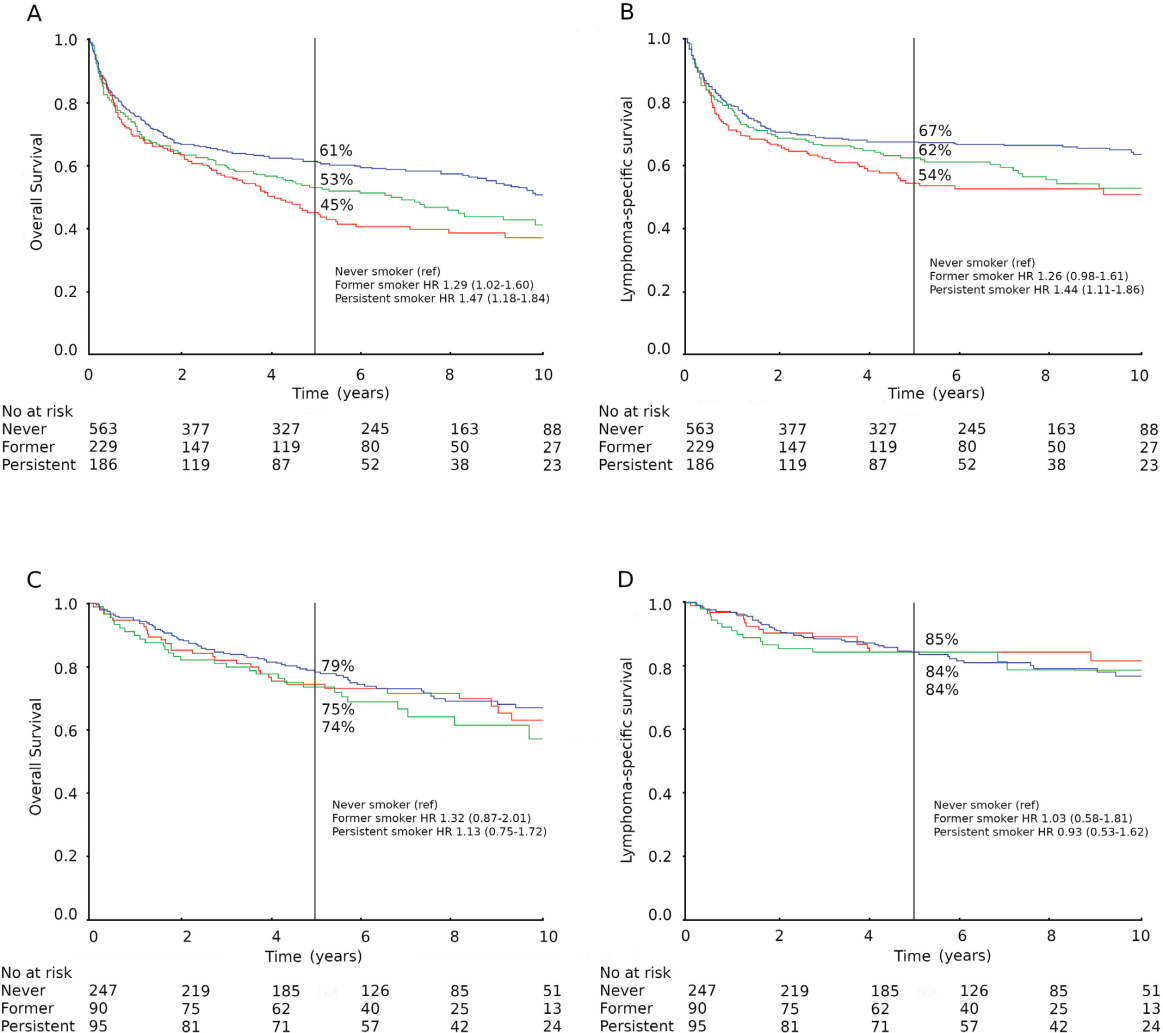
Survival according to smoking status. DLBCL (upper panel A and B) and FL (lower panel C and D), with overall survival (left A and C) and lymphoma-specific survival (right B and D). Never smokers (blue); former smokers (green); persistent smokers (red).

During the median follow-up time of 96 months (interquartile range [IQR] 66–128 months), 662 patients had died, of which 523 died due to lymphoma or leukaemia, followed by cardiovascular causes among smokers (Supplementary Table 5). Five-year OS rates were 61%, 53%, and 45% for never, former, and persistent smokers, respectively (Figure 1A). The 5-year OS rate was 36% for those with unknown smoking status and 52% for the entire DLBCL cohort. For patients initiating systemic treatment (*n* = 762), the OS rates were 70%, 59%, and 53% (Supplementary Figure 1A), respectively. The 5-year OS rate was 46% among those with unknown smoking status and 62% in the whole respective cohort.

The longest median OS (mOS) was observed in never smokers with DLBCL (Figure 1A); mOS was 128, 81, and 50 months for the never, former, and persistent smokers, respectively. mOS was 17 months among those with unknown smoking status and 72 months in the whole DLBCL cohort.

Multivariate Cox regression analysis was performed for OS. After adjustment for sex, age, ECOG performance status, Charlson Comorbidity Index, Ann Arbor stage, and the use of systemic therapy (Table 2), persistent smoking impaired OS independently with HR 1.29 (95% CI 1.05–1.63), and former smoking with HR 1.10 (0.88–1.37) compared to never smokers.

**Table 2:** Multivariable Cox model for overall and lymphoma specific survival in DLBCL.

Covariate	*n*	Median OS, months (5-year rate)	OS multivariable HR (95% CI)	LSS multivariable HR (95% CI)
Smoking status				
Never smoker	563	128 (61%)	Reference	Reference
Former smoker	229	81 (53%)	1.10 (0.88–1.37)	1.10 (0.85–1.42)
Persistent smoker	186	50 (45%)	1.29 (1.05–1.63)	1.25 (0.96–1.63)
Age				
≤ 60 years	257	Not reached (77%)	Reference	Reference
> 60 years	721	57 (49%)	2.31 (1.77–3.01)	2.02 (1.51–2.72)
Sex				
Female	427	119 (60%)	Reference	Reference
Male	551	86 (54%)	1.14 (0.95–1.38)	1.05 (0.85–1.30)
Ann Arbor stage				
I–II	280	155 (72%)	Reference	Reference
III–IV	584	84 (53%)	1.90 (1.51–2.38)	2.33 (1.75–3.11)
Not available	114	8 (33%)	2.52 (1.89–3.43)	3.39 (2.28–4.77)
ECOG PS				
0–1	649	130 (64%)	Reference	Reference
2–4	227	30 (41%)	1.45 (1.19–1.78)	1.49 (1.18–1.89)
Not available	102	21 (43%)	1.37 (1.03–1.81)	1.54 (1.12–2.10)
Charlson Comorbidity Index				
0	447	Not reached (65%)	Reference	Reference
1	233	111 (58%)	1.22 (0.95–1.55)	1.09 (083–1.45)
2–6	298	38 (42%)	1.51 (1.22–1.87)	1.34 (1.05–1.72)
Systemic therapy				
Full-dose ICT 6 cycles	441	Not reached (78%)	Reference	Reference
ICT < 6 cycles or reduced dose	324	48 (46%)	2.49 (1.97–3.14)	2.53 (1.93–3.32)
BSC / palliative radiotherapy only	213	7 (27%)	4.17 (3.25–5.39)	4.44 (3.33–5.93)

BSC: best supportive care only; CI: confidence interval; ECOG PS: Eastern Cooperative Oncology Group performance status; HR: hazard ratio; ICT: immunochemotherapy; LSS: lymphoma-specific survival; OS: overall survival.

A total of 978 patients with known smoking status.

Five-year LSS rates were 67%, 62%, and 54% for never, former, and persistent smokers, respectively (Figure 1B). For patients initiating systemic treatment (*n* = 762), the respective LSS rates were 75%, 66%, and 61% (Supplementary Figure 1B). The longest median LSS (mLSS) was observed in never smokers (Figure 1B). After multivariable adjustment (Table 2), persistent smoking did not reach statistical significance, with HR 1.25 (0.96–1.63), and neither did former smoking, with HR 1.10 (0.85–1.42).

### FL population

FL was diagnosed in 529 patients, with smoking status known for 432 (82%). The datasets for Turku and Tampere were comparable (Supplementary Table 1), but a larger proportion of missing smoking status, stage, BMI, and treatment records were observed in Tampere compared with Turku. Patients from Tampere were older.

In the combined FL cohort, the median age was 67 years, 72% were aged over 60 years at diagnosis, and 47% were male. Ann Arbor stage was III–IV in 67% of the patients (Supplementary Table 2).

Patients with FL and unknown smoking status were comparable to those with known smoking status (Supplementary Table 2), with the exception that best supportive care or missing treatment records were more common among those with unknown smoking status, also information on stage and BMI were more often missing in patients with unknown smoking status.

### Baseline demographics and treatments in FL according to smoking status

A total of 247 (57%) patients with FL were never smokers, 90 (21%) former, and 95 (22%) persistent (Table 3). Persistent smokers were younger at diagnosis compared to never smokers (age over 60 years 55% vs. 75%, and over 75 years 8% vs. 26%). BMI ≤22 kg/m^2^ was most common among persistent smokers. Systemic anticancer treatment was initiated for 166 (69%) never, 57 (65%) former, and 57 (60%) persistent smokers. No statistically significant differences for systemic treatment or radiotherapy proportions were seen based on smoking status (Supplementary Table 4).

**Table 3:** Baseline demographics in FL according to smoking status.

	Never Smoker *n* = 247 (57%)	Former Smoker *n* = 90 (21%)	Persistent Smoker *n* = 95 (22%)	*p*-value
Median age (IQR, range)	67.5 (59**–**76, 29**–**96)	67.8 (61**–**75, 31–88)	60.6 (52**–**69, 19**–**90)	
Age > 60 years	186 (75)	72 (80)	52 (55)	<0.001
Age > 75 years	64 (26)	23 (26)	8 (8)	0.001
Sex: male	107 (43)	51 (57)	49 (52)	0.07
Ann Arbor stage				0.09
I	34 (15)	13 (16)	25 (29)	
II	33 (15)	14 (17)	8 (9)	
III	97 (43)	29 (36)	29 (34)	
IV	61 (27)	25 (31)	23 (27)	
Not available	22	9	10	
ECOG PS				0.76
0–1	180 (83)	72 (86)	68 (85)	
2–4	38 (17)	12 (14)	12 (15)	
Not available	29	6	15	
Charlson Comorbidity Index				0.17
0	146 (59)	47 (52)	43 (45)	
1	46 (19)	23 (26)	26 (27)	
2–6	55 (22)	20 (22)	26 (27)	
BMI				
Median (IQR)	25.9 (23.1**–**28.7)	27.0 (22.5**–**31.5)	25.8 (22.5**–**29.1)	0.41
BMI ≤ 22 kg/m^2^	18 (8)	7 (9)	15 (19)	0.03
BMI > 22 kg/m^2^	201 (92)	71 (91)	66 (81)	
Not available	28	12	10	

BMI: Body mass index; ECOG PS: Eastern Cooperative Oncology Group performance status; IQR: interquartile range.

Statistical testing only with known data.

### Smoking status and survival in FL

During a median follow-up time of 97 months, 147 patients had died, 94 from lymphoma/leukaemia, followed by other cancers or cardiovascular causes (Supplementary Table 5). Sixteen patients (12 never smokers) with FL developed DLBCL during the follow-up. Regardless of smoking status, no difference in 5-year OS rates was observed, 79%, 75%, and 74% for never, former, and persistent smokers, respectively (Figure 1C). Five-year OS rate was 80% for those with unknown smoking status and 77% for all FL patients. mOS or mLSS were not reached, but survival did not differ between smoking status groups (Figure 1C, D).

After multivariate adjustment for sex, age, ECOG performance status, Charlson Comorbidity Index, Ann Arbor stage and treatment, OS, and LSS were unaffected by smoking status (Table 4).

**Table 4:** Multivariable Cox model for overall and lymphoma specific survival in follicular lymphoma.

Covariate	*n*	Median OS, months (5-year rate )	OS multivariable HR (95% CI)	LSS multivariable HR (95% CI)
Smoking status				
Never smoker	247	Not reached (79%)	Reference	Reference
Former smoker	90	Not reached (74%)	1.33 (0.86–2.09)	1.03 (0.57–1.87)
Persistent smoker	95	Not reached (75%)	1.25 (0.81–1.94)	0.99 (0.56–1.77)
Age				
≤60 years	122	Not reached (88%)	Reference	Reference
>60 years	310	150 (73%)	1.77 (1.12–2.77)	1.17 (0.68–1.99)
Sex				
Female	225	155 (79%)	Reference	Reference
Male	207	Not reached (75%)	1.13 (0.79–1.61)	1.21 (0.76–1.91)
Ann Arbor stage				
I–II	127	Not reached (84%)	Reference	Reference
III–IV	264	Not reached (74%)	1.70 (1.08–2.69)	3.48 (1.66–7.32)
Not available	41	118 (71%)	1.80 (0.96–3.35)	2.46 (0.90–6.68)
ECOG PS				
0–1	320	Not reached (81%)	Reference	Reference
2–4	62	72 (55%)	2.42 (1.60–3.67)	2.55 (1.49–4.35)
Not available	50	Not reached (74%)	1.08 (0.63–1.84)	1.32 (0.68–2.57)
Charlson Comorbidity Index				
0	236	Not reached (85%)	Reference	Reference
1	95	150 (80%)	1.63 (1.02–2.60)	2.44 (1.36–4.38)
2–6	101	73 (56%)	2.75 (1.83–4.14)	3.11 (1.79–5.41)
Treatment				
Full-dose ICT 6 cycles	143	Not reached (85%)	Reference	Reference
ICT < 6 cycles or reduced dose	99	128 (66%)	2.25 (1.37–3.69)	1.84 (1.01–3.32)
Rituximab only	45	Not reached (91%)	0.99 (0.48–2.02)	0.66 (0.26–1.66)
Radiotherapy only	47	Not reached (81%)	1.65 (0.77–3.54)	1.62 (0.55–4.80)
No active treatment	98	150 (68%)	1.85 (1.10–3.10)	1.31 (0.68–2.53)

CI: confidence interval; ECOG PS: Eastern Cooperative Oncology Group performance status; HR: hazard ratio; ICT: immunochemotherapy; LSS: lymphoma-specific survival; OS: overall survival.

A total of 432 patients with known smoking status.

## Discussion and conclusion

In the current study, multiple data sources were combined with a unique patient identifier, to demonstrate the fact that patients with DLBCL who continue to smoke have the worst OS and borderline significant LSS compared to never smokers, while former smokers remain in-between. Five-year OS rate was reduced from 61% to 45% and LSS from 67% to 54% among never and persistent smokers, respectively. Persistent smoking independently impaired mOS regardless of age, sex, Ann Arbor stage, ECOG performance status, comorbidities, and administration of systemic therapy.

Previous questionnaire-based studies on tobacco smoking and prognosis of DLBCL included between 120 and 338 patients as compared to 978 patients in the current population-based dataset, where smoking status was retrieved automatically from electronic medical records [17–20]. However, we recognize that smoking status was missing from 22% of the DLBCL patients; this group showed the poorest survival.

The 5-year OS rate of 59% in our study is comparable to 60%–70% observed in previous studies including only patients receiving R-CHOP-based treatment [7, 8, 10]. As compared to the Swedish DLBCL population from 2007 to 2014 [9], 55% of patients had stage III–IV disease (67% in our study), 23% ECOG performance status 2–4 (27% here), and 84% initiated systemic treatment (74% here). These differences could explain the better 5-year OS rate in Sweden (65%). However, it is challenging to put our results into perspective with previous survival data for smokers since 5-year OS rates for smokers with DLBCL were not reported [17–20].

The biological mechanism of how smoking affects DLBCL survival remains unexplored. Biological differences in DLBCL exist. Non-GCB-like DLBCL has an inferior outcome compared to GCB DLBCL [33, 34]. In general, smoking has been associated with a higher prevalence of anti-apoptotic *BCL2* oncogene t(14:18) translocation [35, 36]. Translocations and other rearrangements of BCL2 and MYC significantly worsen the outcome of DLBCL [33, 37], and overexpression of BCL2 is associated with poor prognosis and R-CHOP treatment failure in DLBCL [38]. Thus, the lower survival in smoking DLBCL patients could be explained by these biological factors outside the score of the current study.

Presence of comorbidities also impaired survival in DLBCL (Table 2), and was more prevalent among smokers. Comorbidities could affect dose-intensity, which was not available in the current study, while treatment initiation and completion rates were equal among smokers and never smokers. We also noticed less comorbidities recorded in Tampere, even though poor performance status was noted more often than in Turku. Thus, ICD-10 codes do not necessarily capture all comorbidities. Also the missing smoking status appeared not be random (Supplementary Table 2), and we cannot exclude the possibility that comorbidities are missing non-randomly.

Concerning patients with FL, the effect of smoking on survival has been reported as most profound. Unselected studies with up to 709 FL patients have shown impaired OS when comparing current and never smokers, but 5-year survival rates were not reported [18, 22]. This survival difference was not observed in our study. The FL patients in our study represent a different population that was both older than those in Odutola’s study [22] (median age 67 and 61 years, respectively) and presenting more often with advanced stage III–IV (67% vs. 49%), receiving anticancer therapy more often (64% vs. 45%), and being persistent smokers more often (22% vs. 9%). Odutola et al. [22] did not report the type or amount of chemotherapy or rituximab used. In our cohort, no differences were observed in the proportions of patients receiving systemic treatment or radiotherapy based on smoking status. Previous studies have indicated that patients with FL who are persistent smokers tend to have poorer survival outcomes, particularly among those who smoke the highest quantities of cigarettes [18, 22]. Unfortunately, our study lacked information regarding the number of cigarettes smoked, which may explain the absence of a survival difference based on smoking status in our cohort.

The limitations of our study are common to all registry-based studies: The data in electronic medical records are not always comprehensive; for example, missing smoking status in 18%–22% of patients. Our results could be confounded by a variety of bias including the missing smoking status, other lifestyle factors, and the lack of original treatment intent and ICT dosing intensity [17–19, 29]. International lymphoma prognostication indexes IPI and FLIPI [3, 15] and levels of serum lactate dehydrogenase should also be explored in future studies.

The smoking algorithm used also has limitations, being unable to assess the time frame between cancer diagnoses and the date when smoking status was assessed. We neither had the data on how many cigarettes per day or pack years patients had smoked as that is seldom recorded in the medical records. While never and persistent smokers were identified usually correctly, former smokers remain a challenge for the algorithm [30, 31]. Furthermore, the algorithm is not validated outside Turku.

In conclusion, persistent smoking impaired OS in patients with DLBCL independent of explanatory covariates or treatment completion rate in our population-based retrospective study. Smoking prevention and cessation programs at the national level would be of utmost importance.

## Supplementary Material



## Data Availability

Data can be requested from https://findata.fi/en/.
