# Supplementary material for: Continuous tobacco smoking increases mortality in diffuse large B-cell lymphoma but not follicular lymphoma, a Finnish population-based study
Source: Acta Oncol. 2025 Dec 17;64:44776. doi: 10.2340/1651-226X.2025.44776 (PMC12746680; doi:10.2340/1651-226X.2025.44776)

Supplementary material has been published as submitted. It has not been copyedited, or typeset by Acta Oncologica

Supplementary Table 1. Comparison between cohorts from Turku and Tampere.

	**Turku DLBCL n=570 (45%)**	**Tampere DLBCL n=688 (55%)**	***p*-value**	**Turku FL n=224 (42%)**	**Tampere FL n=305 (58%)**	***p*-value**
Smoking status			<0.001			<0.001
Never smoker	271 (48)	292 (42)		100 (45)	147 (48)	
Former smoker	139 (24)	90 (13)		56 (25)	34 (11)	
Persistent smoker	86 (15)	100 (15)		45 (20)	50 (16)	
Unknown	74 (13)	206 (30)		23(10)	74 (24)	
Age			0.08			0.01
≤60 years	149 (26)	151 (22)		68 (30)	77 (25)	
>60 years	421(74)	537 (78)		156 (70)	228 (75)	
Median age (IQR), years	68 (60–76)	70 (62–78)		66 (58–74)	67 (60–74)	
Sex			0.58			0.14
Female	265 (46)	309 (45)		111 (50)	171 (56)	
Male	305 (54)	379 (55)		113 (50)	134 (44)	
Ann Arbor stage			<0.001			<0.001
I–II	159 (28)	180 (26)		80 (36)	76 (25)	
III–IV	366 (64)	321 (47)		138 (62)	169 (55)	
Not available	45 (8)	187 (27)		6 (3)	60 (20)	
ECOG PS			<0.001			0.006
0–1	336 (59)	458 (67)		175 (78)	208 (68)	
2–4	162 (28)	134 (20)		33 (15)	48 (16)	
Not available	72 (13)	96 (14)		16 (7)	49 (16)	
Charlson Comorbidity Index			<0.001			0.06
0	306 (54)	296 (43)		135 (60)	161 (53)	
1	134 (24)	165 (24)		50 (22)	65 (21)	
2–6	130 (23)	227 (33)		39 (17)	79 (26)	
Systemic therapy			<0.001			<0.001
Full-dose ICT at least 6 cycles*	291 (51)	198 (29)		90 (40)	71 (23)	
Other systemic treatment	149 (26)	185 (27)		75 (34)	103 (34)	
BSC / palliative radiotherapy only	130 (23)	219 (32)		59 (26)	84 (28)	
Unspecified	0 (0)	85 (12)		0 (0)	47 (15)	

Abbreviations: BSC, best supportive care; ECOG PS, Eastern Cooperative Oncology Group performance status; IQR, interquartile range

* Full dose R-CHOP/R-CHOEP or equivalent, including ASCT. For FL also R-bendamustine allowed.

Supplementary Table 2. Baseline demographics and treatments among all patients with DLBCL or FL divided by whether smoking status was known or unknown.

	**DLBCL, all patients n=1258**	**DLBCL, smoking status known n=978 (78%)**	**DLBCL, unknown smoking status n=280 (22%)**	***p*-value**	**FL, all patients n=529**	**FL, smoking status known n=432 (82%)**	**FL, unknown smoking status n=97 (18%)**	***p*-value**
Median age (IQR), years	68.7 (60–77)	67.7 (60–76)	72.4 (65–80)		66.5 (59–74)	66.2 (58–74)	69.9 (63–77)	
Age >60 years	958 (76)	721 (74)	237 (85)	<0.001	384 (72)	310 (72)	74 (76)	0.36
Age >75 years	367 (29)	249 (26)	118 (42)	<0.001	119 (23)	95 (22)	24 (25)	0.56
Sex: male	684 (54)	551 (56)	133 (48)	0.009	247 (47)	207 (48)	40 (41)	0.23
Ann Arbor stage				<0.001				<0.001
I	180 (14)	149 (15)	31 (11)		84 (16)	72 (17)	12 (12)	
II	159 (13)	131 (13)	28 (10)		72 (14)	55 (13)	17 (18)	
III	179 (14)	150 (15)	29 (10)		178 (34)	155 (36)	23 (13)	
IV	508 (40)	434 (44)	74 (26)		129 (24)	109 (25)	20 (21)	
Not available	232 (18)	114 (12)	118 (42)		66 (12)	41 (9)	25 (26)	
ECOG PS				<0.001				0.19
0–1	794 (63)	649 (66)	145 (52)		383 (72)	320 (74)	63 (65)	
2–4	296 (24)	227 (23)	69 (25)		81 (15)	62 (14)	19 (20)	
Not available	168 (13)	102 (10)	66 (24)		65 (12)	50 (12)	15 (16)	
Charlson Comorbidity Index				0.004				0.36
0	602 (48)	447 (46)	155 (55)		296 (56)	236 (55)	60 (62)	
1	299 (24)	233 (24)	66 (24)		115 (22)	95 (22)	20 (21)	
2–6	357 (28)	298 (31)	59 (21)		118 (22)	101 (23)	17 (18)	
Median BMI (IQR), kg/m^2	26.4 (23.3–29.5)	26.6 (23.5-29.7)	25.4 (22.4–28.4)		26.0 (23.0–29.0)	26.0 (23.0-29.0)	26.5 (23.5–29.5)	
BMI ≤22kg/m^2	131 (10)	107 (11)	24 (9)	<0.001	49 (9)	40 (9)	9 (9)	<0.001
BMI >22kg/m^2	902 (72)	741 (76)	161 (58)		396 (75)	338 (78)	58 (60)	
Not available	225 (18)	130 (13)	95 (34)		84 (16)	54 (13)	30 (31)	
Systemic therapy				<0.001				0.02
Full-dose ICT ≥6 cycles*	489 (39)	441 (45)	48 (17)		161 (30)	143 (33)	18 (19)	
Other systemic	334 (27)	267 (27)	67 (24)		178 (34)	144 (33)	34 (35)	
BSC/palliative RT only	349 (27)	213 (22)	136 (49)		143 (27)	107 (25)	36 (37)	
Unspecified	86 (7)	57 (6)	29 (10)		47 (9)	38 (9)	9 (9)	

Abbreviations: BMI, Body mass index; BSC best supportive care; ECOG PS, Eastern Cooperative Oncology Group performance status; IQR, interquartile range; RT, radiation therapy
* Full dose R-CHOP/R-CHOEP or equivalent, including ASCT. For FL also R-bendamustine allowed

Supplementary Table 3: Primary treatment of DLBCL according to smoking status

	Never smoker n=563 (58%)	Former smoker n=229 (23%)	Persistent smoker n=186 (19%)	*p*-value
Systemic therapy initiated				
Yes	447 (79)	183 (80)	135 (73)	0.12
No	116 (21)	46 (21)	51 (27)	
Treatment completion rate				0.25
Full-dose ICT at least 6 cycles	258 (58)	104 (57)	79 (59)	
Other regimens or <6 cycles	148 (33)	70 (38)	49 (36)	
Not available	41 (9)	9 (5)	7 (5)	
Specific regimen				
Full-dose ICT *(R-CHOP21/14, R-CHOEP21/14, R- CEOP, R-DA-EPOCH)*	313 (70)	127 (70)	90 (67)	0.49
Reduced-dose ICT*(R-mini-CHOP, R-COP)*	42 (9)	17 (9)	16 (12)	
Other/unspecified ICT	50 (11)	12 (7)	14 (10)	
Central nervous system protocol	24 (5)	14 (8)	9 (7)	
Chemotherapy only	18 (4)	13 (7)	6 (4)	
Number of cycles				0.76
Full 6 or more	281 (63)	112 (62)	84 (62)	
3-5	65 (15)	29 (16)	22 (16)	
1-2	48 (11)	25 (14)	18 (13)	
Not available	52 (12)	16 (9)	11 (8)	
Radiotherapy for DLBCL				
No radiotherapy	308 (55)	139 (61)	112 (60)	0.45
Consolidation radiotherapy	207 (37)	71 (31)	58 (31)	
Palliative radiotherapy only	48 (9)	19 (8)	16 (9)	

ICT, immunochemotherapy.

Supplementary Table 4. Primary treatment of FL according to different smoking groups.

	**Never smoker n=247 (57%)**	**Former smoker n=90 (21%)**	**Persistent smoker n=95 (22%)**	***p*-value**
Systemic therapy for FL				0.11
Yes, within 3 months	117 (47)	49 (54)	43 (45)	
Yes, after 3 months	54 (22)	10 (11)	14 (15)	
Never	76 (31)	31 (34)	38 (40)	
Treatment completion rate				0.26
Full-dose ICT at least 6 cycles	77 (41)	34 (50)	32 (48)	
Other regimens or <6 cycles	94 (50)	25 (37)	25 (37)	
Not available	19 (10)	9 (13)	10 (15)	
Specific regimen				0.46
Single rituximab	32 (19)	5 (9)	8 (14)	
ICT (R-CHOP, R-BENDA)	131 (77)	51 (86)	46 (81)	
Chemotherapy only	8 (5)	3 (5)	3 (5)	
Radiotherapy for FL				0.08
No radiotherapy	168 (68)	56 (62)	60 (63)	
Curative-intent radiotherapy	42 (17)	26 (29)	25 (26)	
Palliative radiotherapy only	37 (15)	8 (9)	10 (11)	

ICT, immunochemotherapy.

Supplementary Table 5. Causes of death according to ICD-10 codes in the current study. Data available until December 2022. Study permits require minimum of three events in each group, thus some groups not reported (n.r.).

**DLBCL**	Never smoker n=244 (%)	Former smoker n=114 (%)	Persistent smoker n=107 (%)	Smoking status unknown n=197 (%)
Lymphoma/leukemia (C80-95)	187 (77)	91 (80)	83 (78)	162 (82)
Other cancer (C00-72)	13 (5)	6 (5)	n.r.	10 (5)
Neurological (F**, G**)	11 (5)	n.r.	0 (0)	5 (3)
Cardiovascular (I**)	18 (7)	9 (8)	13 (12)	10 (5)
Lung (J**)	n.r.	0 (0)	0 (0)	0 (0)
Other (The rest)	n.r.	n.r.	n.r.	10 (5)

**FL**	Never smoker n=64 (%)	Former smoker n=23 (%)	Persistent smoker n=30 (%)	Smoking status unknown n=30 (%)
Lymphoma/leukemia (C80-95)	47 (73)	16 (70)	17 (57)	14 (47)
Other cancer (C00-72)	4 (6)	0 (0)	6 (20)	3 (10)
Neurological (F**, G**)	3 (5)	n.r.	0 (0)	5 (17)
Cardiovascular (I**)	6 (9)	4 (17)	5 (17)	4 (13)
Lung (J**)	0 (0)	n.r.	n.r.	n.r.
Other (The rest)	4 (6)	0 (0)	n.r.	n.r.

Supplementary Figure 1: **Survival according to smoking status in treated patients with DLBCL**. Overall (right) and lymphoma-specific (left) survival. Subgroup of 762 patients treated at least once with systemic therapy. Never smokers (blue); former smokers (green); persistent smokers (red).

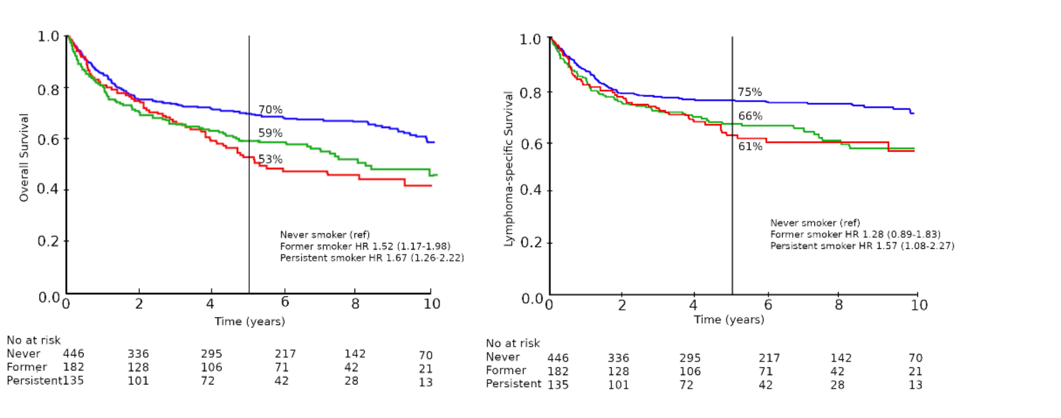

Supplement: Supplementary file 1 [file AO-64-44776-s1.pdf]
